# A Novel Radial Basis Neural Network-Leveraged Fast Training Method for Identifying Organs in MR Images

**DOI:** 10.1155/2020/4519483

**Published:** 2020-05-05

**Authors:** Min Xu, Pengjiang Qian, Jiamin Zheng, Hongwei Ge, Raymond F. Muzic

**Affiliations:** ^1^School of Internet of Things Technology, Wuxi Institute of Technology, Wuxi 214121, China; ^2^School of Internet of Things, Jiangnan University, Wuxi 214122, China; ^3^Jiangsu Key Lab of Media Design and Software Technology, Jiangnan University, Wuxi 214122, China; ^4^School of Artificial Intelligence and Computer Science, Jiangnan University, Wuxi 214122, China; ^5^Department of Radiology and Case Center for Imaging Research, University Hospitals, Case Western Reserve University, Cleveland, OH 44106, USA

## Abstract

We propose a new method for fast organ classification and segmentation of abdominal magnetic resonance (MR) images. Magnetic resonance imaging (MRI) is a new type of high-tech imaging examination fashion in recent years. Recognition of specific target areas (organs) based on MR images is one of the key issues in computer-aided diagnosis of medical images. Artificial neural network technology has made significant progress in image processing based on the multimodal MR attributes of each pixel in MR images. However, with the generation of large-scale data, there are few studies on the rapid processing of large-scale MRI data. To address this deficiency, we present a fast radial basis function artificial neural network (Fast-RBF) algorithm. The importance of our efforts is as follows: (1) The proposed algorithm achieves fast processing of large-scale image data by introducing the *ε*-insensitive loss function, the structural risk term, and the core-set principle. We apply this algorithm to the identification of specific target areas in MR images. (2) For each abdominal MRI case, we use four MR sequences (fat, water, in-phase (IP), and opposed-phase (OP)) and the position coordinates (*x*, *y*) of each pixel as the input of the algorithm. We use three classifiers to identify the liver and kidneys in the MR images. Experiments show that the proposed method achieves a higher precision in the recognition of specific regions of medical images and has better adaptability in the case of large-scale datasets than the traditional RBF algorithm.

## 1. Introduction

Magnetic resonance imaging (MRI) is a new type of high-tech imaging examination fashion in recent years. It has the advantages of no ionizing radiation, no bone artifacts, and multidirectional and multiparameter imaging [1]. Therefore, the generation of an end-to-end intelligent disease diagnosis system based on MRI is an inevitable direction for the development of intelligent medicine. To achieve the goal of effective intelligent medical treatment, this paper studies the classification of abdominal organs based on MRI.

There are many techniques for medical image processing [2–5]. Gordillo et al. [6] divided the existing MR image processing technologies into the following three categories: The first type is threshold-based methods, which classify the segmentation objects (such as pixels) of the MR image by comparing them with different thresholds [7–9]. The second type is region-based methods, which divide several mutually exclusive regions according to preset rules and then categorize pixels with the same attributes into the same region [10, 11]. The third type is pixel-based classification methods, which mainly classify the objects according to the MR multimodal attributes of each pixel. According to whether the training set is labeled or not, they can be subdivided into unsupervised, semisupervised, and supervised methods [12–14].

Of the third type of methods, an artificial neural network as a supervised learning model is applied to the field of medical imaging [15]. It is suitable for image processing without prior distribution assumptions; its application can be divided into three categories: The first type is to apply artificial neural networks directly to MR image processing. Lucht et al. [16] applied a neural network to the dynamic segmentation of MR breast images. Egmont-Petersen et al. [17] used neural networks and multiscale pharmacokinetic features to segment bone tumors in MR perfusion images. Zhang et al. [18] proposed a visual encoding model based on deep neural networks and transfer learning for brain activity measured by functional magnetic resonance imaging. The second type is to use a convolutional neural network or its improved algorithm to segment MR images [19–22]. Khalilia et al. [20] used convolutional neural networks to automatically perform brain tissue segmentation in fetal MRI. Wang et al. [21] used dynamic pixelwise weighting-based fully convolutional neural networks for left ventricle segmentation in short-axis MRI. The third type is to use hybrid neural networks to segment MR images. Glass et al. [23] used a hybrid artificial neural network to segment the inversion recovery image of a normal human brain. Alejo et al. [24] used a hybrid artificial neural network to design an accurate computer-aided method capable of performing region segmentation. Reddick et al. [25] used a hybrid neural network to propose a fully automatic method for segmentation and classification of multispectral MR images.

Based on the review of the above literature, great progress has been made in the use of artificial neural networks for medical image segmentation. However, with the higher resolution requirements of MR images and the increasing size of the dataset, research on fast artificial neural network training for large medical image datasets is still lacking. In response to this phenomenon, this paper proposes the Fast-RBF algorithm, which has fast processing capabilities for large datasets. We applied this method to MRI-based abdominal organ classification and segmentation. The results showed that this method achieved significant results. The main contributions of this paper are as follows:

1. The Fast-RBF algorithm with a large-sample-processing capability is proposed by introducing the *ε*-insensitive loss function and structural risk term and using the core-set principle [26]. This method not only retains the strong nonlinear fitting ability and simple learning rules of RBF artificial neural networks but can also process a large dataset quickly, which improves the processing speed and efficiency.
2. For each abdominal MRI case, we use four MR sequences (fat, water, IP, and OP) and the position coordinates (*x*, *y*) of each pixel as the input of the algorithm. We use three classifiers to identify the liver, kidneys, and other tissues. The proposed algorithm has better adaptability and runs faster in large dataset scenarios than the traditional RBF neural network algorithm.

The remainder of this paper is divided into four parts: Section 2 introduces RBF neural networks and the relationship between RBF neural networks and linear models; Section 3 introduces the Fast-RBF neural network with its large-sample-processing ability; Section 4 verifies the validity of the proposed algorithm on medical image processing; and Section 5 summarizes the full text.

## 2. Related Work

### 2.1. RBF Neural Network

**Figure 1:**
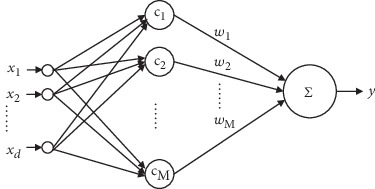
The model of an RBF neural network.

RBF neural networks consist of an input layer, an implicit layer, and an output layer, as shown in Figure 1. Among them, **x**_*i*_ ∈ *R*^*d*^,*y* ∈ *R*, the number of hidden layer nodes is *M*, and the nonlinear mapping *f* : *R*^*d*^⟶*R* is performed by the RBF neural network.

In an RBF neural network, the input layer receives the training samples; the hidden layer node performs a nonlinear transformation through the radial basis function that maps the input space to a new space. If the radial basis function is defined as a Gaussian function, let **c**_*i*_ ∈ *R*^*d*^ denote the center of the Gaussian function and let *δ*_*i*_ represent the kernel width of the Gaussian function. This function can be expressed as

(1) $$\begin{array}{c} \varphi \left(\left\|x-c_{i}\right\|\right)=\exp\left(-\frac{{\left\|x-c_{i}\right\|}^{2}}{\delta_{i}}\right). \end{array}$$

The nodes of the output layer implement a linear weighted combination in this new space. Let *w*_*i*_ be the connection weight of the hidden layer and the output layer and *φ*(•) be the radial basis function; then, the mapping function of *R*^*d*^⟶*R* is

(2) $$\begin{array}{c} y=f\left(x\right)=\overset{M}{\underset{i=1}{\sum }}w_{i}\varphi \left(\left\|x-c_{i}\right\|\right). \end{array}$$

### 2.2. RBF Neural Network and Linear Model

According to the introduction above, the RBF neural network has 3 parameters: the center vector of the radial basis function **c**_*i*_ = [*c*_*i*1_, *c*_*i*2_, ⋯*c*_*id*_]^T^, the kernel width *δ*_*i*_, and the weight of the output layer *w*_*i*_. Among them, **c**_*i*_ and *δ*_*i*_ can be determined by the fuzzy *C*-means (FCM) clustering algorithm [27], and *w*_*i*_ is obtained by the gradient descent learning algorithm. Let *μ*_*ji*_, which is obtained by the FCM clustering algorithm, denote the fuzzy membership of sample **x**_*j*_ for the *i*th class, *n* represent the size of the training sample, and *M* indicate the number of hidden layer nodes; then, the center of the radial basis function **c**_*ik*_ and the kernel width *δ*_*i*_ can be expressed by equations (3) and (4):

(3) $$\begin{array}{c} c_{ik}=\frac{\sum_{j=1}^{n}\mu_{ji}x_{jk}}{\sum_{j=1}^{n}\mu_{ji}}, \end{array}$$

(4) $$\begin{array}{c} \delta_{i}=\frac{\sum_{j=1}^{n}\mu_{ji}{\left\|x_{j}-c_{i}\right\|}^{2}}{\sum_{j=1}^{n}\mu_{ji}}. \end{array}$$

Let ${\tilde{x}}_{i}=\varphi \left(\left\|x-c_{i}\right\|\right)$, *i* = 1, 2, ⋯, *M*.

(5) $$\begin{array}{c} \tilde{x}={\left[{\tilde{x}}_{1},{\tilde{x}}_{2},\cdots ,{\tilde{x}}_{M}\right]}^{\text{T}}. \end{array}$$

The center **c**_*i*_ and the kernel width *δ*_*i*_ of the radial basis function are obtained by equations (3) and (4), the input sample is mapped to the new space *f* : *R*^*d*^⟶*R*^*M*^, and the conversion from the input layer to the hidden layer is a nonlinear mapping.

Let **p** = [*w*_1_, *w*_2_, ⋯*w*_*M*_]^T^; then, the neural network function can be expressed as

(6) $$\begin{array}{c} y=p^{\text{T}}\tilde{x}. \end{array}$$

It can be seen from equation (6) that when the radial basis function hidden layer is estimated, the output of the network can be converted into a linear model.

## 3. Fast-RBF Algorithm

### 3.1. Fast-RBF Principle

First, the *ε*-insensitive loss function corresponding to the RBF linear model is introduced. To minimize the value of the *ε*-insensitive loss function, *ε* is solved as the constraint term of the optimization problem. Then, the structural risk term and the Gaussian kernel are introduced to construct the RBF neural network optimization model with large-sample processing. The specific steps are as follows.

**Step 1 .**

From equations (3) and (4), the values of **c**_*i*_ and *δ*_*i*_ are obtained; then, from equation (5), the model input $\tilde{x}$ is obtained.

**Step 2 .**

Introducing the *ε*-insensitive loss function.

First, the definition of the *ε*-insensitive loss function is given:

**Definition 1 .**

The *ε*-insensitive loss function is defined as [28]

(7) $$\begin{array}{c} L^{\varepsilon }\left(x,y,f\right)={\left|y-f\left(x\right)\right|}_{\varepsilon }=\max\left(0,{\left|y-f\left(x\right)\right|}_{\varepsilon }\right), \end{array}$$

where **x** ∈ *R*^*d*^,*y* ∈ *R*.

For the linear model of equation (6), its corresponding *ε*-insensitive loss function can be expressed as

(8) $$\begin{array}{c} \overset{n}{\underset{i=1}{\sum }}{\left|y_{i}^{o}-y_{i}\right|}_{\varepsilon }=\overset{n}{\underset{i=1}{\sum }}\max\left(0,\left|y_{i}^{o}-y_{i}\right|-\varepsilon \right)=\overset{n}{\underset{i=1}{\sum }}\max\left(0,\left|p^{\text{T}}{\tilde{x}}_{i}-y_{i}\right|-\varepsilon \right), \end{array}$$

where *y*_*i*_^*o*^ represents the neural network output value and *y*_*i*_ represents the real output value.

For equation (8), the constraints of $p^{\text{T}}{\tilde{x}}_{i}-y_{i}<\varepsilon$ and $y_{i}-p^{\text{T}}{\tilde{x}}_{i}<\varepsilon$ are not always satisfied, so the relaxation factors *ξ*_*i*_ and *ξ*_*i*_^∗^ are introduced, and the following constraints can be obtained:

(9) $$\begin{array}{c} \left\{\begin{array}{c} y_{i}-p^{\text{T}}{\tilde{x}}_{i}<\varepsilon +\xi_{i},\;\xi_{i}\geq 0,\\ p^{\text{T}}{\tilde{x}}_{i}-y_{i}<\varepsilon +\xi_{i}^{*},\xi_{i}^{*}\geq 0. \end{array}\right. \end{array}$$

The purpose of this algorithm is to minimize the value of the *ε*-insensitive loss function represented by equation (8). The value of the *ε*-insensitive parameter will directly affect the accuracy of the modeling. Therefore, the parameter *λ* is introduced, and *ε* is used as the constraint term in the optimization problem. Combined with equation (9), the optimization problem can be expressed equivalently as

(10) $$\begin{array}{c} \min2\lambda \varepsilon +\frac{\lambda }{\mu n}\overset{n}{\underset{i=1}{\sum }}\left({\xi_{i}}^{2}+{\xi_{i}^{*}}^{2}\right),\;\text{s}.\text{t}.\left\{\begin{array}{c} y_{i}-p^{\text{T}}{\tilde{x}}_{i}<\varepsilon +\xi_{i},\\ p^{\text{T}}{\tilde{x}}_{i}-y_{i}<\varepsilon +\xi_{i}^{*}, \end{array}\right. \end{array}$$

where the parameter *μ* is the balance factor and *ξ*_*i*_, *ξ*_*i*_^∗^ ≥ 0 is automatically satisfied.

**Step 3 .**

Introducing structural risk items and kernel functions.

A support vector machine is an implementation of the principle of structural risk minimization [28]; the method proposed in this paper learns the implementation method of the support vector machine and introduces a regularization term to minimize the risk in the algorithm structure. The kernel method is an important component of a support vector machine [28], which is used to improve the computational ability of the linear learner. The method proposed in this paper also introduces a kernel function. After introducing the regular term and the kernel function, the optimization problem can be expressed by

(11) $$\begin{array}{c} \underset{p,\varepsilon ,\xi_{i},\xi_{i}^{*}}{\min}{\left\|p\right\|}^{2}+2\lambda \varepsilon +\frac{\lambda }{\mu n}\overset{n}{\underset{i=1}{\sum }}\left({\xi_{i}}^{2}+{\xi_{i}^{*}}^{2}\right),\;\text{s}.\text{t}.\left\{\begin{array}{c} y_{i}-p^{\text{T}}\varphi \left({\tilde{x}}_{i}\right)<\varepsilon +\xi_{i},\\ \begin{array}{c} p^{\text{T}}\varphi \left({\tilde{x}}_{i}\right)-y_{i}<\varepsilon +\xi_{i}^{*},\\ i=1,2,\cdots ,n. \end{array} \end{array}\right. \end{array}$$

**Step 4 .**

Formula derivation.

By introducing the Lagrange multiplier, the Lagrangian function of equation (11) can be expressed as

(12) $$\begin{array}{c} L={\left\|p\right\|}^{2}+2\lambda \varepsilon +\frac{\lambda }{\mu n}\overset{n}{\underset{i=1}{\sum }}\left({\xi_{i}}^{2}+{\xi_{i}^{*}}^{2}\right)+\overset{n}{\underset{i=1}{\sum }}\alpha_{i}\left(y_{i}-p^{\text{T}}\varphi \left({\tilde{x}}_{i}\right)-\varepsilon -\xi_{i}\right)+\overset{n}{\underset{i=1}{\sum }}\alpha_{i}^{*}\left(p^{\text{T}}\varphi \left({\tilde{x}}_{i}\right)-y_{i}-\varepsilon -\xi_{i}^{*}\right). \end{array}$$

The matrix form of the corresponding dual problem of equation (12) is

(13) $$\begin{array}{c} \left\{\max\left[\alpha^{T}\;\alpha^{*T}\right]\left[\begin{array}{c} \frac{2}{\lambda }y\\ -\frac{2}{\lambda }y \end{array}\right]-\left[\begin{array}{cc} \alpha^{T} & \alpha^{*T} \end{array}\right]\tilde{K}\left[\begin{array}{c} \alpha \\ \alpha^{*} \end{array}\right],\;\text{s}.\text{t}.\left[\begin{array}{cc} \alpha^{T} & \alpha^{*T} \end{array}\right]1=1,\;\alpha ,\alpha^{*}\geq 0\right., \end{array}$$

where *α*, *α*^∗^ are the Lagrange coefficients and $\tilde{K}$ is the kernel function. They are

(14) $$\begin{array}{c} y=\left[\begin{array}{c} y_{1}\\ \vdots \\ y_{n} \end{array}\right],\\ \alpha =\left[\begin{array}{c} \alpha_{1}\\ \vdots \\ \alpha_{n} \end{array}\right],\\ \alpha^{*}=\left[\begin{array}{c} {\alpha_{1}}^{*}\\ \vdots \\ {\alpha_{n}}^{*} \end{array}\right],\\ \tilde{K}=\left[\tilde{k}\left({\tilde{x}}_{i},{\tilde{x}}_{j}\right)\right]=\left[\begin{array}{cc} K+\frac{\mu n}{\lambda }I & -K\\ -K & K+\frac{\mu n}{\lambda }I \end{array}\right], \end{array}$$

where K is the Gaussian kernel function.

The values of the variables obtained by the solution are

(15) $$\begin{array}{c} \left\{\begin{array}{c} p=\lambda \overset{n}{\underset{i=1}{\sum }}\left(\alpha_{i}-\alpha_{i}^{*}\right)\varphi \left({\tilde{x}}_{i}\right),\\ \begin{array}{c} \xi_{i}=\alpha_{i}\mu n,\\ \xi_{i}^{*}=\alpha_{i}^{*}\mu n. \end{array} \end{array}\right. \end{array}$$

In addition, because ∑_*i*=1_^*n*^(*α*_*i*_ + *α*_*i*_^∗^) = 1, *μ* = ∑_*i*=1_^*n*^(*ξ*_*i*_ + *ξ*_*i*_^∗^)/*n*.

**Step 5 .**

Prediction.

The prediction function is shown in the following equation:

(16) $$\begin{array}{c} y=p^{T}\varphi \left({\tilde{x}}_{\text{test}}\right)=\lambda \overset{n}{\underset{i=1}{\sum }}\left(\alpha_{i}-\alpha_{i}^{*}\right)\varphi^{T}\left({\tilde{x}}_{i}\right)\varphi \left({\tilde{x}}_{\text{test}}\right)=\lambda \overset{n}{\underset{i=1}{\sum }}\left(\alpha_{i}-\alpha_{i}^{*}\right)\tilde{K}\left({\tilde{x}}_{i},{\tilde{x}}_{\text{test}}\right). \end{array}$$

If it is used for classification,

(17) $$\begin{array}{c} y=\mathrm{sign}\left(p^{T}\varphi \left({\tilde{x}}_{\text{test}}\right)\right). \end{array}$$

If *y* > 0, it belongs to the positive class, and if *y* < 0, it belongs to the negative class.

It can be seen from this section that the algorithm proposed in this paper is a quadratic programming problem.

### 3.2. The Center-Constrained MEB Problem

In 2002, Bădoiu and Clarkson proposed a minimum enclosing ball (MEB) (1 + *ξ*)-approximation algorithm based on the core set in the literature [26]. The algorithm uses a subset of the input set, which is called the core set, to solve the optimization problem. The algorithm can obtain the same good approximation results as the original input set to improve the efficiency of the algorithm. Tsang et al. [29] suggested that the MEB problem is related to many kernel problems. Eligible quadratic programming (QP) problems can be solved quickly by the core-set algorithm. The following section briefly introduces the center-constrained minimum enclosing ball (CC-MEB) algorithm. Next, we will introduce the relationship between the proposed algorithm and CC-MEB and implement the fast algorithm proposed in this paper.

Given the training sample *S* = {*φ*(**x**_*i*_)}_*i*=1_^*m*^, where **x**_*i*_ ∈ *R*^*d*^ and *φ* is the kernel mapping associated with a given kernel **K**, adding one dimension *δ*_*i*_ to each *φ*(**x**_*i*_) forms a set *S* = {(*φ*^*T*^(**x**_*i*_), *δ*_*i*_)}_*i*=1_^*m*^. By setting the coordinate of the last one-dimensional center point to be 0, that is, the CC-MEB's coordinate is [**c**, 0], then the optimization problem of finding the smallest CC-MEB that contains all the samples in the set *S* is

(18) $$\begin{array}{c} \left\{\underset{c,R}{\min}R^{2},\;\text{s}.\text{t}.{\left(\varphi \left(x_{i}\right)-c\right)}^{2}+{\delta_{i}}^{2}\leq R^{2}\right., \end{array}$$

where *i* = 1, 2, ⋯*m*.

Let Δ = [*δ*_1_^2^, ⋯,*δ*_*m*_^2^]^*T*^ ≥ 0; then, the matrix form of the corresponding dual problem of equation (18) is

(19) $$\begin{array}{c} \left\{\underset{\beta }{\max}\beta^{T}\left(\mathrm{diag}\left(K\right)+\Delta \right)-\beta^{T}K\beta ,\;\text{s}.\text{t}.\beta \geq 0,\beta^{T}1=1\right., \end{array}$$

where the kernel matrix is

(20) $$\begin{array}{c} K_{m\times m}=\left[k\left(x_{i},x_{j}\right)\right]=\left[\varphi^{T}\left(x_{i}\right)\varphi \left(x_{j}\right)\right]. \end{array}$$

Using the optimal solution **β** to obtain the values for the radius *R* and center point **c**,

(21) $$\begin{array}{c} \left\{\begin{array}{c} R=\sqrt{\beta^{T}\left(\mathrm{diag}\left(K\right)+\Delta \right)-\beta^{T}K\beta }\\ c=\overset{m}{\underset{i=1}{\sum }}\beta_{i}\varphi \left(x_{i}\right). \end{array}\right. \end{array}$$

The formula for the distance from any point to the center point is

(22) $$\begin{array}{c} {\left\|c-\varphi \left(x_{l}\right)\right\|}^{2}+\delta_{l}^{2}={\left\|c\right\|}^{2}-2{\left(K\beta \right)}_{l}+k_{ll}+{\delta_{l}}^{2}. \end{array}$$

Because **β**^*T*^1 = 1, any real number *η* is added to equation (19), which does not affect the value of **β**. The original dual form is changed to

(23) $$\begin{array}{c} \left\{\underset{\beta }{\max}\beta^{T}\left(\mathrm{diag}\left(K\right)+\Delta -\eta 1\right)-\beta^{T}K\beta ,\;\text{s}.\text{t}.\beta \geq 0,\beta^{T}1=1,\Delta \geq 0\right.. \end{array}$$

Reference [29] pointed out that any QP problem that satisfies equation (23) can be regarded as an CC-MEB problem, which can be solved by the core-set fast algorithm

### 3.3. Relationship between Fast-RBF and CC-MEB

Equation (13) is the QP form of Fast-RBF. Let $\tilde{\alpha }={\left[\begin{array}{cc} \alpha^{T} & \alpha^{*T} \end{array}\right]}^{T}$; then,

(24) $$\begin{array}{c} \Delta =-\mathrm{diag}\left(\tilde{K}\right)+\eta 1+\frac{2}{\lambda }\left[\begin{array}{c} y\\ -y \end{array}\right], \end{array}$$

where the real number *η* should be large enough so that Δ ≥ 0. Thus, equation (13) can be written as follows:

(25) $$\begin{array}{c} \left\{\begin{array}{cc} \underset{\tilde{\alpha }}{\max} & {\tilde{\alpha }}^{T}\left(\mathrm{diag}\left(\tilde{K}\right)+\Delta -\eta 1\right)-{\tilde{\alpha }}^{T}\tilde{K}\tilde{\alpha }\\ s.t. & {\tilde{\alpha }}^{T}1=1. \end{array}\right. \end{array}$$

This form is equivalent to the CC-MEB problem from equation (23), and the problem can be solved using the core-set fast algorithm [29].

According to formula (25), the center of sphere **c** can be calculated as $c=\sum_{i=1}^{2*n}{\tilde{\alpha }}_{i}\tilde{\varphi }\left({\tilde{x}}_{i}\right)$. In the formula, when *i* = 1, ⋯, *n*, then $\tilde{\varphi }\left({\tilde{x}}_{i}\right)=\varphi \left({\tilde{x}}_{i}\right)$; when *i* = *n* + 1, ⋯, 2*n*, then $\tilde{\varphi }\left({\tilde{x}}_{i}\right)=-\varphi \left({\tilde{x}}_{i}\right)$, and the derivation is available:

(26) $$\begin{array}{c} c=\overset{2*n}{\underset{i=1}{\sum }}{\tilde{\alpha }}_{i}\tilde{\varphi }\left({\tilde{x}}_{i}\right)=\overset{n}{\underset{i=1}{\sum }}\alpha_{i}\varphi \left({\tilde{x}}_{i}\right)+\overset{n}{\underset{i=1}{\sum }}\alpha_{i}^{*}\left(-\varphi \left({\tilde{x}}_{i}\right)\right)=\overset{n}{\underset{i=1}{\sum }}\left(\alpha_{i}-\alpha_{i}^{*}\right)\varphi \left({\tilde{x}}_{i}\right). \end{array}$$

Therefore, the value of **p** in equation (15) is **p** = *λ ***c**.

### 3.4. The Implementation of Fast-RBF

**Figure 2:**
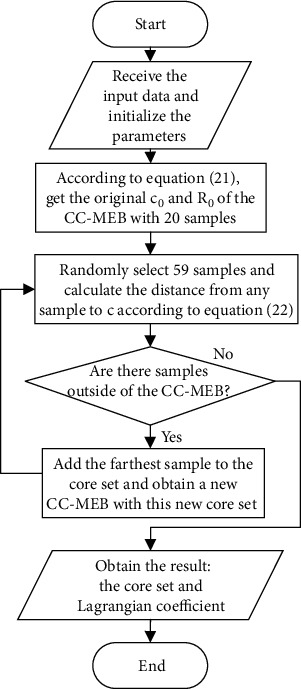
Flow chart of the algorithm.

Algorithm 1 describes the steps of the Fast-RBF algorithm, and the flow chart is shown in Figure 2.

## 4. Experimental Results and Analysis

In this paper, the effectiveness of the proposed method is verified by comparing it with the traditional RBF algorithm on MR images. The experiment is divided into two stages: the parameter optimization stage and the modeling stage. In the parameter optimization stage, the grid search method is used to obtain the optimal parameters of each algorithm based on the training set. In the modeling stage, the training set is modeled using optimal parameters, and the test set is used to obtain the performance of each algorithm.

The experiment is verified from the following four aspects:

1. Verify that the size of the core set of the Fast-RBF algorithm is much smaller than the training set's scale, which can speed up the modeling time of the algorithm
2. Verify that the prediction capability of the Fast-RBF algorithm is comparable to the prediction capability of the RBF algorithm
3. Verify that the modeling time of the Fast-RBF algorithm on large datasets is much smaller than that of the RBF algorithm

For the experimental environment, the operating system is Windows 10; the processor is an Intel i5 2.71 GHz CPU; the memory is 8 GB; and the main application software is MATLAB R2015a.

### 4.1. Experimental Preparation

**Figure 3:**
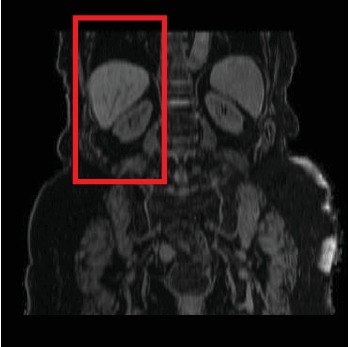
Areas to be identified.

The use case in this section is from MRI scans of five subjects recruited by the University Hospitals Cleveland Medical Center Institutional Review Board. Before the experiment, a block diagram is first used to frame the area to be identified, as shown in Figure 3. Next, we train and test the data of region of interest in abdominal organ map. The experiment is to classify the liver and kidneys of the region of interest in the abdominal organ map.

For each case, we extract the local textural features from four different types of abdominal 3D MR images, namely, fat, water, in-phase (IP), and opposed-phase (OP), as the input of the algorithm. Noise cannot be avoided in these actual data, and this noise will affect the final image recognition effect. Therefore, this paper adopts the method proposed in [30, 31] to design a convolution kernel as shown in Table 1, preprocesses the experimental data, and implements feature extraction.

In addition, we also consider the pixel spacing of the MR images and adopt the grid division strategy. Let (*x*, *y*) represent the position information of the pixel. That is, we combine the features that we extracted and obtain a six-dimensional feature.

### 4.2. Experimental Method

We define the liver of the region of interest in abdominal MR images as class A, the kidneys as class B, and other tissues as class C. Therefore, this is a multiclassification problem. We train “liver (class A)-kidney (class B),” “liver (class A)-other tissue (class C),” and “kidney (class B)-other tissue (class C)” to obtain three classification results; the final result is then determined by voting. The voting method is as follows:

Let *A* = *B* = *C* = 0.

The classification is (*A*, *B*) if it belongs to *A*, and *A* = *A* + 1; otherwise, *B* = *B* + 1.

The classification is (*A*, *C*) if it belongs to *A*, and *A* = *A* + 1; otherwise, *C* = *C* + 1.

The classification is (*B*, *C*) if it belongs to *B*, and *B* = *B* + 1; otherwise, *C* = *C* + 1.

The final sample belongs to the class with the largest values of *A*, *B*, and *C*.

The classification accuracy is used to measure the performance of the algorithm.

(27) $$\begin{array}{c} \text{prediction accuracy}=\frac{\text{the number of correctly classified test samples}}{\text{total number of test samples}}. \end{array}$$

### 4.3. Experimental Results

Cases 1-4 contain a total of 59,904 data points. We randomly selected 10,000 data points, 20,000 data points, 30,000 data points, 40,000 data points, 50,000 data points, and 59,904 data points for training. Case 5, which contains 16,896 data points, was used as the test set. The experiment was repeated 10 times for each training set size to verify the advantages of the proposed method.

#### 4.3.1. Core Set of the Fast-RBF Algorithm

**Figure 4:**
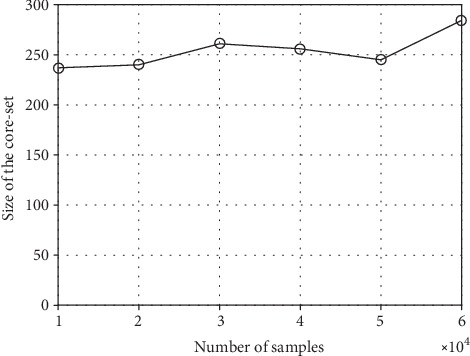
The size of the core set at different sample sizes.

Figure 4 shows the average values of the total number of core-set samples for the three classifiers at different training set sizes. Figure 4 shows that the total number of core sets is between 240 and 300, which is much smaller than the sample size. Replacing all the samples with the core sets in the model construction step will greatly improve the operational efficiency.

#### 4.3.2. Prediction Ability of the Fast-RBF Algorithm

**Figure 5:**
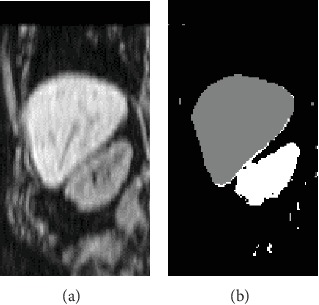
Test results. (a) Original picture. (b) Organ classification results.

It can be seen from Table 2 that both algorithms can achieve a good generalization performance. However, with the increase in the amount of training data, the RBF algorithm requires more samples to participate in the modeling step, so it is more constrained. When the data size exceeds 30,000 data points, it can no longer be solved. The Fast-RBF algorithm uses core-set technology to solve the problem. Key samples are added to the core set one by one, and the average size of the core set does not exceed 300, so it can process a larger dataset and can achieve a generalization ability comparable to that of the RBF algorithm. The organ classification results are shown in Figure 5.

#### 4.3.3. Time Performance of the Fast-RBF Method

Table 3 shows that the modeling time required by the two algorithms has a stable growth with increasing sample size. When the size of the dataset is 30,000 data points, the average modeling time of the RBF algorithm is 7,580 seconds, while the average time of the Fast-RBF algorithm is 15.2609 seconds. The modeling time of the Fast-RBF algorithm is much smaller than that of the RBF algorithm. In addition, when the size of the dataset is more than 30,000 data points, the RBF algorithm will not run.

### 4.4. Experimental Conclusion

It is known from experiments that the Fast-RBF algorithm can be used for organ recognition in MR images. The advantage of the proposed algorithm is that it requires far less modeling time than the RBF algorithm in large datasets under the premise of ensuring the prediction accuracy. The algorithm has strong practicability.

## 5. Conclusion

Our studies are based on MRI of challenging body sections of the abdomen. We proposed the Fast-RBF algorithm, which is suitable for the rapid training of a large dataset. By introducing the *ε*-insensitive loss function, learning the structural risk term and kernel method of the support vector machine, and using the core-set principle, the proposed algorithm can meet the needs of large sample sizes. This method can quickly process large datasets and is suitable for medical image processing.

The method proposed in this paper is a supervised learning method. The training samples need to be labeled, and the workload of data preparation is large. In the future, we will further study the semisupervised abdominal image recognition method in which only a small number of class labels are needed to achieve image processing.

## Figures and Tables

**Algorithm 1:**
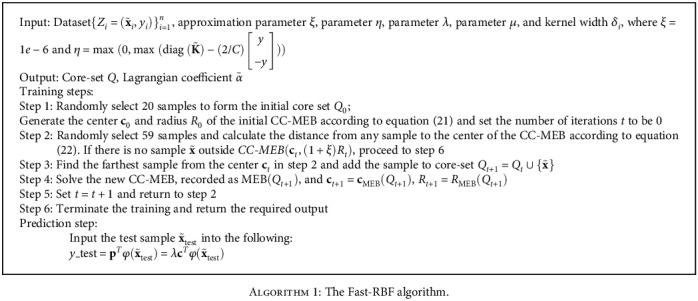
The Fast-RBF algorithm.

**Table 1:** Convolutional kernel CK_3×3_.

0.1	0.1	0.1
0.1	0.2	0.1
0.1	0.1	0.1

**Table 2:** Prediction accuracy and standard deviation of the two algorithms at different dataset sizes.

Size of the dataset	Prediction accuracy and standard deviation	
	RBF	Fast-BRF
10,000	0.9345 ± 0.0083	0.9458 ± 0.0153
20,000	0.9389 ± 0.0063	0.9551 ± 0.0132
30,000	0.9381 ± 0.0104	0.9496 ± 0.0098
40,000	—	0.9467 ± 0.0111
50,000	—	0.9432 ± 0.0112
59,904	—	0.9552 ± 0.0066

**Table 3:** Average modeling time and standard deviation of the two algorithms under different data sizes.

Size of the dataset	Modeling time and standard deviation of each method (s)	
	RBF	Fast-BRF
10,000	210.5813 ± 3.5134	10.1719 ± 7.0177
20,000	737.1344 ± 7.1357	12.8016 ± 4.0126
30,000	7.58*E* + 03 ± 164.0596	15.2609 ± 8.2559
40,000	—	16.5953 ± 9.1518
50,000	—	17.5953 ± 9.1983
59,904	—	22.9781 ± 7.0587

## Data Availability

Data sharing is not available for our study, as the experimental data were afforded by our collaboration partners at the University Hospitals Cleveland Medical Center, OH, USA. Without permission, we cannot share any of our data with others.

## Additional Points

*Human and Animal Rights*. The experimental data are from five subjects recruited by the University Hospitals Cleveland Medical Center Institutional Review Board. We used these data to be compatible with the board's requirements.
